# Central nervous system manifestations of monogenic autoinflammatory disorders and the neurotropic features of SARS-CoV-2: Drawing the parallels

**DOI:** 10.3389/fped.2022.931179

**Published:** 2022-08-10

**Authors:** Thomas Renson, Lorraine Hamiwka, Susanne Benseler

**Affiliations:** ^1^Division of Rheumatology, Department of Pediatrics, Cumming School of Medicine, University of Calgary, Calgary, AB, Canada; ^2^Division of Nephrology, Department of Pediatrics, Cumming School of Medicine, University of Calgary, Calgary, AB, Canada; ^3^Department of Internal Medicine and Pediatrics, Ghent University Hospital, Ghent, Belgium; ^4^Alberta Children's Hospital Research Institute, Cumming School of Medicine, University of Calgary, Calgary, AB, Canada

**Keywords:** autoinflammation, central nervous system, monogenic autoinflammatory disease, COVID-19, vasculitis

## Abstract

Central nervous system (CNS) involvement in monogenic autoinflammatory disorders (AID) is increasingly recognized and can be life threatening. Therefore, a low threshold to consider CNS disease should be maintained in patients with systemic inflammation. Hyperinflammation is also a key feature of severe acute COVID-19 and post COVID-19 entities such as multisystem inflammatory syndrome in children. Like AID, COVID-19 patients can present with severe CNS involvement. The impact of COVID-19 on AID and CNS involvement in particular is still obscure, nevertheless dreaded. In the current review, we synthesize the spectrum of CNS manifestations in monogenic AID. We explore common pathophysiological and clinical features of AID and COVID-19. Moreover, we assess the impact of immune dysregulation associated with SARS-CoV-2 infections and post COVID-19 hyperinflammation in AID. The striking commonalities found between both disease entities warrant caution in the management of AID patients during the current pandemic.

## Introduction

The term autoinflammation was coined by McDermott et al. in 1999 to describe the phenotype of inherited periodic fevers characterized by seemingly unprovoked spikes of hyperinflammation, but without evidence of autoantibody and self-reactive antigen-specific T cell involvement (1, 2). Autoinflammatory disorders (AID) arise from aberrant innate immune responses, which differentiates them from autoimmune diseases wherein chiefly the adaptive immune system is dysregulated. The monogenic AID are a heterogeneous group of diseases covering a broad clinical spectrum, often associated with recurrent episodes of fever, organ-specific inflammation including serositis, arthritis and skin rash, and markedly raised acute phase reactants. Central nervous system (CNS) involvement is rarely on the forefront of the clinical phenotype, albeit is increasingly recognized in AID patients (3, 4).

The genesis of severe acute respiratory coronavirus 2 (SARS-CoV-2) and its associated coronavirus disease 2019 (COVID-19) in December 2019 caused a shift in the way we care for our patients (5). Numerous questions and concerns arose, not only regarding the susceptibility for severe COVID-19 of patients treated with immunosuppressive drugs, but also about the possible impact on the disease course of the underlying inflammatory rheumatic condition. Moreover, COVID-19 triggered atypical clinical presentations of established diseases that are still dreaded to this day (6, 7). Scarce data exist regarding the repercussions of COVID-19 on the course of monogenic AID. Similarly, data on the impact of COVID-19 on CNS disease in AID are limited.

The aims of this review were therefore to describe the spectrum of monogenic AID and their respective CNS manifestations, to evaluate the impact of COVID-19 on AID patients, and to assess the neurotropic potential of SARS-CoV-2. These results enable us to hypothesize on the potential impact of COVID-19 on autoinflammatory CNS disease by drawing several parallels between COVID-19, autoinflammation and the CNS.

## Spectrum of monogenic autoinflammatory diseases

Since the identification of the mutation causing familial Mediterranean fever (FMF) 25 years ago (8), numerous advances in genomic screening tools have enormously enhanced our understanding of the concept and pathophysiology of monogenic AID. In this heterogeneous group of diseases, genetic mutations dysregulate the normal innate immune system homeostasis. The aberrant activation of specific immunological pathways leads to an increased expression of innate immunity cytokines. These recurrent episodes of hyperinflammation delineate the characteristic clinical phenotype of AID.

### The inflammasome

Inflammasomopathies comprise a class of AID characterized by aberrantly activated interleukin (IL)-1β-stimulating multimeric protein hubs, i.e., inflammasomes (2). As the most prevalent entity and one of the first AID described, FMF, a NLRP3 inflammasomopathy caused by mutation of the MEFV gene encoding pyrin, serves as the archetype of monogenic AID (8, 9). FMF is characterized by recurrent episodes of fever and polyserositis (10). Pyrin is also involved in the pathophysiology of hyperimmunoglobulinemia D syndrome (HIDS), a monogenic AID caused by MVK mutations (11, 12). The clinical phenotype of HIDS mainly encompasses recurrent fever, abdominal pain, skin rash, and joint involvement (13). In keeping with the concept of inflammasomopathies, cryopyrin-associated periodic syndromes (CAPS) are caused by gain-of-function (GOF) mutations in the NLRP3 gene (14–16). Three distinct clinical entities had previously been described, but were found to reflect the severity spectrum of autosomal dominant CAPS: familial cold autoinflammatory syndrome (FCAS), Muckle-Wells syndrome (MWS), and chronic infantile neurological, cutaneous, and articular syndrome (CINCA) (14–16). Notwithstanding initially described as separate diseases, FCAS, MWS and CINCA are now considered to be part of a disease continuum with several overlapping features. CINCA has the most menacing clinical phenotype, distinguished by the neonatal onset of persisting fever, urticarial skin rash and unremitting elevation of acute phase reactants, followed by a hypertrophic arthropathy and severe CNS disease (17). The rare tumor necrosis factor receptor-associated periodic syndrome (TRAPS), initially called familial Hibernian fever when first described in 1982 in 16 members of an Irish family, is associated with mutations in the tumor necrosis factor receptor superfamily member 1A (TNFRSF1A) gene (18–20). TRAPS is a hyperinflammatory multisystem disorder characterized by a broad and heterogeneous clinical spectrum with recurrent fever as its cardinal symptom (21). Finally, pyogenic arthritis, pyoderma gangrenosum, and acne (PAPA) syndrome, caused by PSTPIP1 gene mutations, is characterized by the constellation of arthritis, pyoderma gangrenosum and severe cystic acne (22).

### Type 1 interferon axis

Type 1 interferonopathies are a group of monogenic AID distinguished by an inherent activation of the type 1 interferon (IFN) pathway (23, 24). Aicardi-Goutières syndrome (AGS) is caused by mutations in TREX1, IFIH1, ADAR, RNASEH2A, RNASEH2B, RNASEH2C, or SAMHD1 (25). Its clinical phenotype chiefly comprises an early-onset encephalopathy (23, 25). Stimulator of interferon genes (STING)-associated vasculopathy with onset in infancy (SAVI) is a type 1 interferonopathy caused by GOF mutations in TMEM173 (26). SAVI is particularly characterized by vasculitis with prominent skin features and interstitial lung disease. Other type 1 interferonopathies fall outside the scope of this review.

### Other monogenic autoinflammatory disorders

Several monogenic AID outside the concepts of inflammasomopathies and interferonopathies are acknowledged. Deficiency of adenosine deaminase-2 (DADA2), caused by mutations in ADA2, is regarded as a mimic of polyarteritis nodosa as it typically presents with a small to medium-sized vessel vasculitis leading to early-onset stroke (27, 28). Haploinsufficiency of A20 (HA20), associated with mutations in the TNFAIP3 gene, is an early-onset autoinflammatory syndrome often mimicking the clinical spectrum of Behçet's disease (BD), including recurrent oral and genital ulcers, polyarthritis, skin involvement, and gastrointestinal manifestations (29). Considering A20 is a vigorous inhibitor of the NFκB pathway, HA20 leads to upregulation of NFκB-associated proinflammatory cytokines (29, 30). The NFκB pathway is also upregulated in patients with granulomatous inflammatory disease/Blau syndrome, an early-onset autoinflammatory granulomatous disease associated with NOD2 mutations (31, 32). The clinical spectrum typically encompasses the triad of skin manifestations, joint involvement, and eye disease (31, 33). Deficiency of IL-1 receptor antagonist (DIRA) is an IL1RN gene-associated AID resulting in neonatal pustulosis and sterile osteomyelitis (34). Recently, an intriguing novel class of adult-onset AID was described by the term VEXAS (vacuoles, E1 enzyme, X-linked, autoinflammatory, somatic) syndrome, associated with somatic mutations in UBA1 (35). Patients typically develop an inflammatory phenotype in late adulthood characterized by fever, bone marrow dysplasia, neutrophilic skin rash, pulmonary inflammation, vasculitis, chondritis, and the presence of distinctive vacuoles in myeloid and erythroid precursor cells.

## Central nervous system manifestations of autoinflammatory disorders

### The spectrum of central nervous system involvement

**Figure 1:**
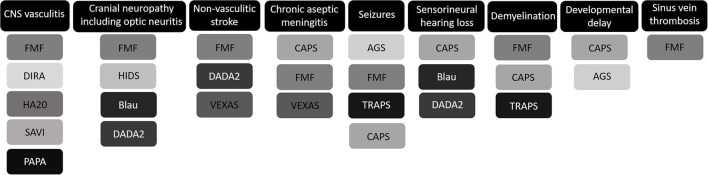
Graphic overview of the most prominent central nervous system (CNS) manifestations of monogenic autoinflammatory disorders. FMF, familial Mediterranean fever; DIRA, deficiency of IL-1 receptor antagonist; HA20, haploinsufficiency of A20; SAVI, stimulator of interferon genes associated vasculopathy with onset in infancy; PAPA, pyogenic arthritis, pyoderma gangrenosum, and acne syndrome; HIDS, hyperimmunoglobulinemia D syndrome; DADA2, deficiency of adenosine deaminase-2; VEXAS, vacuoles, E1 enzyme, X-linked, autoinflammatory, somatic syndrome; CAPS, cryopyrin-associated periodic syndromes; AGS, Aicardi-Goutières syndrome; TRAPS, tumor necrosis factor receptor-associated periodic syndrome.

Neurological features are generally not a cardinal feature of AID, but CNS manifestations are increasingly recognized and can be prominent and life threatening (Figure 1). CINCA is notorious for its CNS involvement, whereas this is rarely observed in the other CAPS subtypes. Chronic aseptic meningitis, leading to raised intracranial pressure, hydrocephaly, brain atrophy, and papilledema if untreated, is the most frequent CNS manifestation of CINCA, observed in 26% of patients (17). Furthermore, sensorineural hearing loss secondary to persisting cochlear inflammation and seizures were repeatedly reported in case series (4, 17, 36). If untreated the CNS involvement in CINCA may lead to severe intellectual disability. Chronic papilledema may lead to optic nerve atrophy and progressive loss of vision (17).

AGS is also characterized by prominent CNS involvement. It typically involves an early-onset progressive sub-acute encephalopathy associated with developmental delay and seizures, and the occurrence of basal ganglia calcifications and leukodystrophy on CNS imaging (23, 25, 37). Increased IFN levels in the cerebrospinal fluid have been detected in AGS patients. These levels exceed serum concentrations, implying a role for intrathecal IFN synthesis (23, 25). Aseptic meningitis has not only been described in CINCA patients, but also in VEXAS syndrome and FMF patients (38–40). CNS involvement in FMF is generally rare. Nevertheless, a broad spectrum of co-occurrences of CNS manifestations in patients with FMF have been reported in multiple case reports and case series, including optic neuritis, pseudotumor cerebri, ataxia, sinus vein thrombosis, seizures, demyelinating lesions (see below), ischaemic stroke, and CNS vasculitis (see below) (3, 41–49). Optic neuritis has also been described in a 43-year-old woman with an atypical presentation of HIDS (50). Nonetheless, CNS manifestations rarely occur in HIDS patients.

Cranial neuropathies are the hallmark of CNS involvement in granulomatous inflammatory disease/Blau syndrome (sixth cranial nerve palsy) (33). In addition, these can be seen in patients with DADA2 (optic neuritis and other cranial neuropathies) (51, 52), and those with NLRP3 low penetrance variants (53). Similarly to CINCA and MWS, Blau syndrome and DADA2 can present with sensorineural hearing loss (33, 54, 55). In addition to CINCA, AGS and FMF, seizures have also been reported in 1% of the TRAPS population from the Eurofever/EUROTRAPS registry (21). Refractory epilepsy is the hallmark of febrile infection-related epilepsy syndrome (FIRES) (56). Although the pathophysiology of FIRES remains obscure, it possibly involves a NLRP3 inflammasome-mediated IL-1-driven inflammation (57). In keeping with this hypothesis, anakinra (IL-1 inhibition) can be efficacious in some patients (58).

### Central nervous system vasculitis

CNS vasculitis, a rare manifestation of autoinflammation, has been described in FMF, DIRA, HA20, SAVI, and PAPA syndrome (29, 34, 59–63). Invasion of the vessel wall of the cerebral vasculature by distinct immune cells can lead to stenosis and occlusion inducing ischaemic stroke, but can also cause endothelial dysfunction leading to vascular fragility, extravasation of blood and hemorrhagic stroke.

Notwithstanding the association between FMF and several systemic vasculitides (including IgA vasculitis, BD and PAN) is widely acknowledged (64–66), the link with CNS vasculitis is uncommon. IgA vasculitis, BD, and PAN are all associated with the occurrence of cerebral vasculitis (67–70). Presumably, MEFV gene mutations leading to an altered pyrin induce a pro-inflammatory state, resulting in endothelial dysfunction and a higher susceptibility for vasculitis in FMF patients (59, 71). Luger et al. reported the occurrence of a brain stem infarction in a 34-year-old genetically confirmed FMF patient (59). Digital subtraction angiography revealed an asymptomatic occlusion of a temporal M2-branch and an insular M3-branch of the left middle cerebral artery. Leptomeningeal biopsy confirmed the presence of vasculitis with perivascular infiltration of macrophages and cytotoxic T cells. In the initial case series of nine DIRA patients by Aksentijevich et al., one patient presented with CNS vasculitis/vasculopathy (34). In one of the first HA20 case series, two patients (13%) with CNS vasculitis were described (one of them diagnosed on brain imaging, the other on frontal lobe biopsy) (62). Moreover, the occurrence of CNS vasculitis was reported in the initial report on HA20 (29). In 2016, a group from Stanford University published the case of a young PAPA syndrome patient with a subarachnoid hemorrhage caused by a ruptured posterior cerebral artery dissection (61). An underlying CNS vasculitis was presumed based on the extensive arterial irregularities detected on magnetic resonance angiography. CNS involvement has been reported in only one SAVI patient (63). A 5-year old patient developed blurred vision, headache and hallucinations 14 months after disease onset. Brain MRI revealed bilateral stenosis of the middle cerebral arteries and posterior cerebral arteries, presumably reflecting CNS vasculitis. Interestingly, TREX1 mutations, associated with a spectrum of autoinflammatory/autoimmune diseases including AGS, familial chilblain lupus and monogenic lupus (72), have also been linked to CNS vasculitis (73, 74). Chronic type 1 IFN signaling is the main inflammatory pathway involved in TREX1 enzyme dysfunction (75). Moreover, cerebrovascular stenosis and stroke, presumably reflecting CNS vasculitis, have been described in four patients with SAMHD1 mutations (76), which are also associated with AGS. SAMHD1 mutations have also been identified as risk factors for non-vasculitic stroke (77, 78).

Systemic vasculitis frequently occurs in DADA2 patients. However, it is rarely the cause of stroke, a cardinal manifestation of DADA2 (79). Instead, DADA2 is mainly associated with lacunar and hemorrhagic strokes caused by endothelial dysfunction and vascular frailty. Strikingly, one case report described spinal cord ischemia in a DADA2 patient (52). Non-vasculitic stroke has also been associated with FMF and VEXAS syndrome (40, 47, 49).

Kawasaki disease (KD) is the archetype of diseases linking autoinflammation and vasculitis, in which a cytokine storm causes vascular wall edema and fragility. Nonetheless, the association between monogenic AID and CNS vasculitis/vasculopathy remains obscure and mainly based on case reports, making it difficult to assume a causal relationship. The pro-inflammatory and pro-thrombotic state often associated with autoinflammation may nevertheless imply a higher vulnerability to vasculitis and vasculopathy in general.

### The autoinflammation—demyelination connection: The NLRP3 inflammasome as common denominator?

Strikingly, an association has been suggested between AID and multiple sclerosis (MS), a chronic inflammatory CNS disease characterized by relapsing/remitting attacks of white matter demyelination. Several case series have reported the co-occurrence of FMF and MS in patients (45, 46, 80–82). Of note, Elhani et al. reported 23 out of 24 FMF patients (96%) having inactive disease at time point of MS onset (46). Only one patient had a diagnosis of MS prior to the onset of FMF. Interestingly, some authors described a higher prevalence of heterozygous MEFV mutations in MS patients compared to healthy subjects (83–85). In contrast, other studies did not detect this difference (86–88). It remains therefore elusive whether FMF predisposes to MS (and/or vice versa), demyelinating lesions wrongfully diagnosed as MS are a rare manifestation of FMF, or the described association between both diseases relies solely on coincidence. Kümpfel et al. reported that 24% of MS patients with two additional TRAPS-compatible symptoms (including arthralgia/arthritis, urticarial rash, and severe fatigue) carry the TRAPS-associated low-penetrance TNFRSF1A R92Q mutation (89). The carrier frequency of the respective mutation in MS patients without two additional TRAPS symptoms did not significantly differ compared to controls. TNFRSF1A has emerged as a susceptibility locus for MS in a meta-analysis of genome-wide association studies and in an independent replication cohort (90). The higher prevalence of TNFRSF1A variant carriers in MS patients compared to healthy controls was subsequently confirmed in a Belgian and an Argentinian population (91, 92). The co-occurrence of MS and CAPS has been described in a recent case report (93). MS has also been associated with NLRP3 low-penetrance mutations.

The association between AID and MS may reflect common pathophysiologic mechanisms. Caspase-1 expression is strikingly increased in the oligodendrocytes of MS lesions (94). Furthermore, caspase-1 inhibition prevented cytokine-induced apoptosis in a human oligodendroglial hybrid cell line. NLRP3 gene expression is substantially increased in an MS-like demyelination and neuroinflammation mouse model (95). Moreover, mice lacking the NLRP3, IL-18, or CASP1 gene showed reduced demyelination. Increased IL-1β levels have been detected in the CSF of active MS patients compared to inactive MS patients and other neurological diseases (96). Additionally, the expression levels of IL-1β and caspase-1 were significantly increased in peripheral blood mononuclear cells of MS patients compared to healthy controls (97–99). Collectively, these studies suggest a role for NLRP3 inflammasome-mediated IL-1β pathway activation in the pathophysiology of MS. Thus, MS and AID including FMF, CAPS, and TRAPS, may exhibit a genetic and a pathophysiologic linkage.

## The impact of COVID-19 on central nervous system autoinflammation

### COVID-19 in patients with autoinflammatory disorders

Despite the scarce evidence on the impact of COVID-19 on AID patients and CNS autoinflammation in particular, important lessons can be learned. Collectively, COVID-19 as a viral trigger did not seem to dramatically dysregulate innate immunological pathways in AID patients (Table 1). Of the 151 AID patients with COVID-19 described in several cohorts, six patients are deceased (4.0%) (100–102, 104–109). The French RMD COVID-19 cohort assessed SARS-CoV-2 infection in 694 patients with an inflammatory rheumatic condition (101). Of those, only 13 patients were children. Twelve children (92%) had a benign COVID-19 disease course and none developed severe disease. Twenty-seven patients in this cohort had AID, of which three were children: two had FMF, one had cryopyrinopathy. Five out of 27 AID patients in this cohort (19%) had a severe COVID-19 disease course. Of note, AID emerged as a risk factor for severe COVID-19 (aOR 7.88; 95% CI: 1.39–37.05). Another French study by Bourguiba et al. assessed COVID-19 in a cohort of 342 FMF patients [median age 33 (IQR 17–87)] (102). Twenty-seven patients (7.9%) had a confirmed SARS-CoV-2 infection. Of those, seven (26%) needed hospital admission and three (11%) had acute respiratory distress syndrome requiring intensive care unit admission, mechanical ventilation and hemodialysis. None of the patients with severe COVID-19 were <40 years old. In a study by Sozeri et al., 87 children with rheumatic disease (median age 12 years; 35% had AID) and suspected COVID-19 were analyzed (103). Fifty-six (64%) patients from this retrospective cohort were hospitalized. Welzel et al. reported a case series of four AID patients with COVID-19: two CAPS patients aged 12 and 14 years, one FMF patient aged 15 years, and one unclassified (u)AID patient aged 34 years (104). All four patients, especially the adolescents, experienced a mild COVID-19 disease course, albeit both CAPS patients experienced increased disease activity a few weeks post-infection. In contrast, Sozeri et al. reported no disease flare in 113 children (mean age 13 ± 4.7 years) with an inflammatory rheumatic condition (of which 39 had AID) following COVID-19 (105).

**Table 1:** COVID-19 disease course in patients with autoinflammatory disorders (AID) varies greatly.

**Study reference**	**AID patients with COVID-19**	**Anti-inflammatory AID treatment**	**COVID-19 disease course**					
			**Asymptomatic or mild**	**Moderate**	**Severe**	**Hospital admission**	**ICU**	**Deceased**
Haslak et al. (100)	7 (6 FMF, 1 PFAPA)	6 colchicine	2 Asympt. (29%)	NL	NL	1	NL	0
Florence et al. (101)	27 (15 PFS, 5 Still, 3 sJIA, 4 other)	NL	13 Mild (48%)	9 (33%)	5 (19%)	NL	NL	4 (15%)
Bourguiba et al. (102)	27 (FMF)	26 Colchicine, 1 TNFi, 1 ILi	NL	NL	NL	7 (26%)	3 (11.1%)	2 (7%)
Sozeri et al. (103)	30 (FMF/HIDS/CAPS)	8 Biologic agent	NL	NL	NL	NL	NL	NL
Welzel et al. (104)	4 (2 CAPS, 1 FMF, 1 uAID)	3 Canakinumab, 1 methotrexate, 1 colchcine	4 (100%)	0	0	0	0	0
Sozeri et al. (105)	39 (21 FMF, 5 CAPS, 4 HIDS, 4 DADA2, 3 CRMO, 1 IRP)	NL	NL	NL	NL	NL	NL	0
Meyts et al. (106)	7 (3 FMF, 3 AGS, 1 uAID)	FMF: none, canakinumab, colchicine uAID: post-RTX, steroids AGS: JAKi	5 (71%)	NL	NL	2 (29%)	0	0
Sengler et al. (107)	18 (11 FMF, 2 NLRP3-associated, 3 TRAPS, 1 PFAPA, 1 DADA2)	NL	0 Asympt.	NL	NL	0	0	0
Peet et al. (108)	14	NL	1 Asympt. (7%)	NL	NL	2 (14%)	0	0
Tobor-Swietek et al. (109)	8 (6 CAPS, 2 Schnitzler)	NL	8 (100%)	0	0	0	0	0

“Not listed” (NL): no information was given or this data could not be extracted from the paper because the AID patients were described among a cohort of patients with other inflammatory rheumatic conditions. ICU, intensive care unit; FMF, Familial Mediterranean fever; PFAPA, periodic fever, aphthous stomatitis, pharyngitis, adenitis syndrome; PFS, periodic fever syndromes (not specified); Still, adult-onset Still's disease; sJIA, systemic-onset juvenile idiopathic arthritis; CAPS, cryopyrin-associated periodic syndromes; uAID, unspecified autoinflammatory disorder; HIDS, hyperimmunoglobulinemia D syndrome; DADA2, deficiency of adenosine deaminase-2; CRMO, chronic recurrent multifocal osteomyelitis; IRP, idiopathic recurrent pericarditis; AGS, Aicardi-Goutières syndrome; TRAPS, tumor necrosis factor receptor-associated periodic syndrome; Schnitzler, Schnitzler's syndrome.

COVID-19 may trigger Multisystem Inflammatory Syndrome in Children (MIS-C), a hyperinflammatory immune dysregulation characterized by fever, the frequent occurrence of KD-like mucocutaneous manifestations, and a risk of circulatory shock (110–112). Considering the associated innate immune cell activation with cytokine storm and the overlap in clinical features, MIS-C is often difficult to differentiate from relapse in AID patients. This was demonstrated in a recent case series of three FMF patients with MIS-C, of which all had fever and abdominal pain obscuring the diagnosis (113). Laboratory features that can aid in discriminating MIS-C from AID include thrombocytopenia, lymphocytopenia, and high n-terminal pro hormone B-type natriuretic peptide and D-dimer levels.

Altogether, this data do not substantiate a higher susceptibility of AID patients for severe COVID-19 [without prejudice considering the French RMD COVID-19 cohort study (101)], despite the frequent use of immunosuppressive drugs. Some authors reported an AID relapse following COVID-19, albeit qualitative comparative studies are lacking to assess whether this reflects a significant association. Of note, none of the mentioned studies reported the impact of COVID-19 or MIS-C on CNS involvement in AID patients.

### The central nervous system link: The neurotropic features of SARS-CoV-2

An impact of COVID-19 on autoinflammatory CNS involvement has never been described. Delineating the possible impact of SARS-CoV-2 on the brain may teach us how COVID-19 and CNS autoinflammation are interrelated. A continuously expanding spectrum of CNS manifestations of acute COVID-19 are recognized, notwithstanding the upper respiratory system is the main port-of-entry of SARS-CoV-2 and respiratory symptoms are its predominant disease manifestations. CNS involvement may include headache, confusion and cognitive impairment, and more severe, cerebral venous sinus thrombosis, ischemic stroke, cerebral and subarachnoid hemorrhage, meningitis/encephalitis, posterior reversible encephalopathy syndrome, acute disseminated encephalomyelitis, and acute necrotizing hemorrhagic encephalopathy (114–116). MIS-C can also present with CNS manifestations, which may differ from the CNS involvement in acute COVID-19. Interestingly, coronaviruses have been reported to be associated with MS since the 1980s (117, 118). Multiple studies reported the isolation of coronaviruses from the CNS of MS patients, in addition to the detection of coronavirus antigens and RNA (117–122). Moreover, a chronic infection with coronavirus has shown to trigger inflammatory demyelinating lesions in an MS-like mouse model (123).

Several mechanisms have been suggested as to how SARS-CoV-2 can exert its neurotropic functions in the CNS. Direct CNS invasion implies a breach of the blood-brain barrier. Nevertheless, ACE2, the main docking receptor of SARS-CoV-2, is not expressed in the endothelial cells lining the cerebral vasculature and only in low levels in the choroid plexus and neocortical neurons (124–126). SARS-CoV-2 may enter the CNS by infecting peripheral neurons (the olfactory and vagus nerve in particular) and use the axonal transport machinery to travel upstream, a mechanism called neuronal retrograde dissemination (114, 118). Finally, the aforementioned cytokine storm associated with SARS-CoV-2 may harm the brain without direct viral neuroinvasion (114). Several pro-inflammatory cytokines and chemokines including CXCL10, CCL7, and CCL11 were shown to be upregulated in the CSF until at least 7 weeks post-infection in a mouse model of mild respiratory SARS-CoV-2 infection (127). In these mice, impaired hippocampal neurogenesis, decreased oligodendrocytes and myelin loss were observed following infection. Of interest, human patients with long-COVID and associated cognitive impairment (i.e., “brain fog”) following a mild respiratory SARS-CoV-2 infection had similar elevated CCL11 plasma levels compared to patients without cognitive impairment (127). These studies demonstrate that even a mild respiratory SARS-CoV-2 infection may harm the brain without direct viral neuroinvasion.

### Commonalities in the pathophysiology of COVID-19 and autoinflammation

The pathophysiology of (severe) COVID-19 and AID are strikingly interrelated. Both diseases give rise to an aberrant activation of the innate immune system. Severe COVID-19 is characterized by a cytokine storm, a surge in pro-inflammatory cytokines and chemokines including TNFα, IL-6, IL-1β, IL-2R/CD25, IL-10, IP-10, IL-2, IL-8, IL-17, G-CSF, GM-CSF, MCP1, and CCL3 (5, 128–130). Interestingly, patients admitted to the intensive care unit (ICU) displayed higher plasma levels of IL-2, IL-7, IL-10, G-CSF, IP10, MCP-1, CCL3, and TNFα compared to non-ICU patients (5). COVID-19 alters innate immune responses by viroporin-mediated NLRP3-inflammasome activation, increasing IL-1β expression (131). Strikingly, acute COVID-19 escapes immune surveillance by counteracting the antiviral IFN signaling pathway, consequently augmenting its own viral replication and further stimulating aberrant immune processes (128, 132). Bastard et al. detected autoantibodies against type 1 IFN in 101 out of 987 patients (10.2%) with life-threatening COVID-19 pneumonia (132). These autoantibodies represented an inborn error of type 1 IFN immunity, predisposing individuals to severe COVID-19. Moreover, van der Made et al. reported a case series of four young males with severe COVID-19 having X-linked loss-of-function variants of TLR7 with downregulated type 1 IFN signaling (133). Of interest, an overwhelming cytokine storm is also observed in MIS-C (6, 111, 112). Children with MIS-C typically present with clinical and biochemical features of KD and macrophage activation syndrome (MAS); both KD and MAS have an autoinflammatory origin.

Thus, on the one hand an overlap exists between the pathophysiology of COVID-19 and AID such as NLRP3-inflammasomopathies. These commonalities may explain why AID patients treated with biologicals such as IL-1 inhibition, TNFα inhibition, and IL-6 inhibition are not at greater risk of severe COVID-19. On the contrary, these drugs may even protect AID patients from severe COVID-19 by opposing the associated cytokine storm. On the other hand, COVID-19 is characterized by an antagonized type 1 IFN axis, an important active pathway in autoinflammatory interferonopathies. This specific feature of SARS-CoV-2 might render AID patients treated with janus kinase (JAK) inhibitors more susceptible to severe COVID-19. Nevertheless, this is not observed in case reports, although data on COVID-19 in AID patients on JAK inhibition is scarce (106). Moreover, BenevolentAI's knowledge graph, an artificial intelligence platform, identified baricitinib, a JAK 1 and 2 inhibitor, as a potential drug to reduce SARS-CoV-2's ability to infect lung cells (134). Baricitinib has shown in subsequent randomized clinical trials to reduce recovery time, especially in patients on high-flow oxygen or non-invasive ventilation, and to reduce all-cause mortality in hospitalized adult COVID-19 patients (135, 136). The efficacy of JAK inhibition in (severe) COVID-19 is presumably explained by counteraction of the type 2 IFN response.

### Connecting the dots: The relation between central nerve system autoinflammation and COVID-19

AID and acute COVID-19 are strikingly interconnected regarding their pathophysiology and CNS involvement. Notwithstanding COVID-19 does not dramatically derail the immune system in AID patients, the impact of COVID-19 on autoinflammatory CNS disease remains obscure considering the paucity of data. However, several interesting parallels between AID with CNS involvement and COVID-19 can be drawn. Although not the primary disease manifestation, both AID and COVID-19 may present with similar CNS involvement as described in this review. CNS disease is increasingly recognized in both disease entities. Interestingly, AID and several coronaviruses have been associated with the onset of MS. AID and COVID-19 are also linked on a pathophysiological level. In concordance with AID, COVID-19 triggers an aberrant innate immune response and associated cytokine storm. COVID-19 provokes an NLRP3-mediated increase in IL-1β expression, similar to the NLRP3-inflammasomopathies. Furthermore, children may present with MIS-C following SARS-CoV-2 infection. MIS-C shares pathophysiological, clinical, and biochemical features with the autoinflammatory conditions KD and MAS, which complicates the diagnosis of MIS-C in AID patients. Collectively, these findings may suggest a higher susceptibility for severe COVID-19 in AID patients, assumably also regarding CNS involvement, and a potential risk of subsequent flare of AID and/or CNS autoinflammation. Most monogenic AID patients receive drugs inhibiting pivotal cytokines in the pathophysiology of severe COVID-19, which in turn may act protective. SARS-CoV-2 has the ability to escape innate immune surveillance by opposing type 1 IFN signaling. The beneficial effect of JAK inhibitors including baricitinib in severe COVID-19 presumably reflects inhibition of the type 2 IFN response, which may override the possible negative effect of muting the type 1 IFN pathway. Several additional knowledge gaps remain. Notwithstanding COVID-19 and MIS-C are acute, episodic diseases, CNS inflammation may result in longstanding damage and/or symptoms (e.g., the above described “brain fog”). Moreover, the chronic immune dysregulation in AID patients may predispose to a prolonged and/or recurrent COVID-19/MIS-C related CNS involvement. Larger qualitative longitudinal studies are therefore necessary to evaluate the true impact of COVID-19 in AID patients, assessing severity of COVID-19 disease course and the prevalence of possible COVID-19 triggered AID relapse. Moreover, the long-term implications for the brain should be taken into account in these patients, as CNS involvement in both diseases is still often overlooked and underemphasized.

## Author contributions

TR performed the main literature search and wrote the first draft of the manuscript. LH and SB supervised this process and reviewed the draft version. All authors were involved in outlining the concept and framework of this review article and contributed to the article and approved the submitted version.

## Conflict of interest

The authors declare that the research was conducted in the absence of any commercial or financial relationships that could be construed as a potential conflict of interest.

## Publisher's note

All claims expressed in this article are solely those of the authors and do not necessarily represent those of their affiliated organizations, or those of the publisher, the editors and the reviewers. Any product that may be evaluated in this article, or claim that may be made by its manufacturer, is not guaranteed or endorsed by the publisher.
